# Efficacy of different doses of atorvastatin treatment on serum levels of 8-hydroxy-guanin (8-OHdG) and cardiac function in patients with ischemic cardiomyopathy

**DOI:** 10.12669/pjms.311.5840

**Published:** 2015

**Authors:** Yu Jin, Chunguang Qiu, Qiangsun Zheng, Ling Liu, Zhiqiang Liu, Yi Wang

**Affiliations:** 1 Yu Jin, Department of Cardiology,Wuhan General Hospital of Guangzhou Military Command, Wuhan 430070, Hubei Province, China.; 2 Chunguang Qiu, Department of Cardiology, The First Affiliated Hospital of Zhengzhou University, Zhengzhou 450052, Henan Province, China.; 3 Qiangsun Zheng, Department of Cardiology, Tangdu Hospital of the Fourth Military Medical University, Xi'an 700038, Shanxi Province, China.; 4 Ling Liu, Third Department of Cardiology, Xinxiang Central Hospital, Xinxiang 453000, Henan Province, China.; 5 Zhiqiang Liu, Third Department of Cardiology, Xinxiang Central Hospital, Xinxiang 453000, Henan Province, China.; 6 Yi Wang, Third Department of Cardiology, Xinxiang Central Hospital, Xinxiang 453000, Henan Province, China.

**Keywords:** Atorvastatin, Ischemic cardiomyopathy, 8-Hydroxrguanin, DNA damage, Oxidative stress

## Abstract

**Objective:**

To compare the efficacy of 40 mg and l0 mg atorvastatin on serum levels of 8-Hydroxy-Guanin (8-OHdG) and the cardiac function in patients with ischemic cardiomyopathy (ICM).

**Methods:**

One hundred twenty three hospitalized ICM patients and 120 healthy controls were included in this study. All subjects were randomly divided into two groups: 10 mg/d atorvastatin group (n=62) and 40 mg/d atorvastatin group (n=61). Serum levels of C-reactive protein (CRP), creatine kinase, glutamic-pyruvic transaminase, lipids and B-type natriuretic peptide (BNP) were tested in all subjects both at the initial phase and the terminal phase of this study. Adverse drug reaction events were recorded in this study. Echocardiographic method was applied to compare the cardiac function before and after treatment in the double blind study. Serum 8-OHdG levels were tested by enzyme-linked immunosorbent assay (ELISA) before and after treatment, and the results in atorvastatin treatment groups were compared with the healthy controls.

**Results:**

Serum 8-OHdG levels in ICM patients were significantly higher than that in normal control groups (p<0.05). There was significant difference of Serum 8-OHdG levels in 40 mg/d atorvastatin group (p<0.05), but was no significant difference in 10 mg/d atorvastatin group before and after the treatment. The 8-OHdG level in 40 mg/d atorvastatin group was significantly lower than that in 10 mg/d atorvastatin group before the treatment as well as after the treatment (p<0.05). The systolic and diastolic function improved significantly in 40 mg/d atorvastatin group before and after treatment, as well as in comparison with 10 mg/d atorvastatin group (p<0.05).

**Conclusion:**

Serum 8-OHdG possibly plays an important role in the pathogenesis of ICM. Atorvastatin is safe and effective in ICM treatment; furthermore atorvastatin which also has independent lipid lowering effect, is significantly better in the dose of 40 mg/day.

## INTRODUCTION

More and more evidence has found that oxidative stress is closely linked to the coronary artery heart disease (CAHD) and its complications. Dyslipidosis is the dominant cause of atherosclerosis, which is also a facilitator of increasing reactive oxygen, and will lead the oxidative stress phenomenon, endothelial dysfunction, and cardiovascular complications. 8-Hydroxrguanin is the most typical oxidative damage product, and commonly used as a sensitive biomarker in the oxidative stress and oxidative DNA damage assessment.

As an antiatherosclerotic drug, atorvastatin can appreciably lower the incidence of cardiovascular disease, and is extensively used in the secondary prevention of CAHD.^1^ However, there are very few studies to evaluate other functions of atorvastatin except for blood lipid concentration reduction, and the effect of different doses of atorvastatin usage that related to oxidative stress and cardiac function among ischemic Cardiomyopathy (ICM) patients. By studying the different doses of atorvastatin used in patients, this study was designed to compare the effect of atorvastatin on cardiac function by applying 40 mg/d and 10 mg/d treatment doses respectively among ICM patients, as well as the influence of 8-OHdG serum levels and the amelioration of myocardial ischemia.

## METHODS

***Subjects:*** Total 123 hospitalized ICM patients from the department of Cardiology in the first affiliated hospital of Zhengzhou university were enrolled in this study during March 2010 and March 2013, which included 79 males and 44 females, and their ages ranged from 30 to 80 years, with the average age of (60.88±9.97) years. 123 patients were randomly divided into two groups according to the order of hospital admission date, 10 mg/d atorvastatin treatment group (Group A) included 62 patients, 38 males (61.3%) and 24 females (38.7%), and the average age was (62.14±9.94) years; while 40 mg/d atorvastatin treatment group (Group B) included 61 patients, 41 males (67.2%) and 20 females (32.8%), and the average age was (61.28±10.12) years. Meanwhile, 120 healthy people in physical examination departments were recruited as normal controls (Group C) including 78 males (65.0%) and 42 females (35.0%) with the average age of (62.28±10.12) years. Statistical test showed that there were no significant differentiations between Group A, Group B and Group C in the aspect of sex and age.

This study was conducted in accordance with the declaration of Helsinki and with approval from the Ethics Committee of the First Affiliated Hospital of Zhengzhou University. Written informed consent was obtained from all participants and participants were followed up for one year. Patients in Group A and Group B could receive other medications during atorvastatin treatment on the basis of their disease conditions. During the period of one year follow-up, the incidence of constipation, abdominal distension, indigestion, abdominal pain, myalgia, polymyositis, rhabdomyolysis and drug induced hepatitis were recorded. The diagnostic criterion of drug induced hepatitis was Alanine transaminase (ALT) over 80 IU/L. Two patients in 10mg/d treatment group and one patient in 40mg/d treatment group were lost in the follow-up. The diagnostic criteria of ICM including: (1) Have definite evidence of CAHD such as angina, myocardial infarction, proved over 50% angiostegnosis of the anterior branch, circumflex artery, one or above one right coronary artery by applying coronarography inspection. The method and standard of coronarography was Judkins method. Conventional projection position for left, right coronary artery angiography, and a quantitative analysis in coronary artery stenosis was done by using the angiography image manipulation system; (2) The left ventricular Diastolic internal diameter>50mm; (3) LVEF<45%, ICM patients with III-IV phases of New York Heart Function Assessment (NHYA). The exclusion criteria of ICM including: (1) Patients in congestive heart failure induced by coronary heart disease complicated with severe mitral regurgitation, interventricular septal defect, ventricular aneurysm and arrhythmia or patients with cardiomegaly and heart failure induced by other pathogenesis; (2) Patients clearly diagnosed as chronic inflammation, vasculitis, active infection of other physical parts, thyroid disease, hepatopathy, Autoimmune diseases, stroke, renal failure, lung disease and patients under one year of life expectancy.

***General data collection and laboratory indexes:*** We recorded the general data from all participants including age, sex, body weight, blood pressure; BMI (Body Mass Index), the history of high blood pressure, diabetes and CAHD, the history of coronary artery stent implantation, cigarette smoking, alcohol consumption, hyperlipemia etc. Both in the initial and terminal phase of the study, all study subjects were required to test the C-reactive protein (CRP), total cholesterol (TC), triglyceride (TG), low-density lipoprotein (LDL-C), high-density lipoprotein (HDL-C), serum uric acid (SUA), creatine kinase (CK), alanine aminotransferase (ALT) and brain natriuretic peptide (BNP). Alcohol intake or high fat diet was forbidden 24 hour before blood sampling. 4 ml non-anticoagulative ulnar vein blood was sampled after 12 hour fasting. Bloods was centrifuged as 3000 rpm for 20 min within one hour after sampling, and then segregated the supernatant liquid, which was partially used to test the former mentioned biochemical indexes by automatic biochemical analyzer (Hitachi Ltd, Tokyo, Japan), and partially stored in a -20°C refrigerator for further test of 8-OHdG. Enzyme-linked immunosorbent assay (ELISA) was applied to measure the human serum 8-OHdG, following the instructions in the test kit (Trevigen, Gaithersburg, MD, USA).

***Doppler echocardiography for heart function detection:*** We adopted the Philips iE33 Color Doppler ultrasonography (Philips, Amsterdam, Netherland), the transducer frequency was 2.5 MHz, and ultrasonography was operated by a professional in the Department of Ultrasonography. We measured the left ventricular end diastolic dimension (LVEDD), left ventricular end systolic dimension (LVESD), left ventricular ejection fraction (LVEF) and left ventricular mass index (LVMI) at the initial and terminal phase of this study, and measured the mitral valve early diastolic blood flow peak velocity (E) and late diastolic blood flow peak velocity (A) from the heart apical four chamber section. Average of three readings was recorded.

***Statistical analysis:*** SPSS11.0 software (SPSS Inc, Chicago, IL, USA) was used to execute statistical analysis, quantitative data was shown as ±s, and qualitative data was shown as percentage, t-test was applied to compare the differences in quantitative data, while chi-square test was applied to test the differences in qualitative data. P value less than 0.05 indicated statistically significant difference.

## RESULTS

***General data:*** At the initial phase of this study, there were no statistical significances (p>0.05) between 10mg/d treatment group (group A) and 40mg/d treatment group (group B) in the aspect of age, sex, weight, hypertension, diabetes, history of coronary artery stent implantation, cigarette smoking, family history of CAHD, biochemical index, 8-OHdG, BMI, results of cardiac function and cardiac color Doppler ultrasonic examination. The 8-OHdG level of IGM patients both in group A and group B were significantly higher than people in normal control group (p<0.05, Table-I).

***Comparison of 8-OHdG, CRP, BNP, LDL-C, TC, CK, ALT and cardiac function before and after the study:*** At the terminal phase of this study, the 8-OHdG, CRP, BNP, LDL-C and TC in 40 mg/d treatment group was lower than 10 mg/d treatment group, the difference was statistically significant (p<0.05), and these indexes in 40 mg/d treatment group decreased significantly after treatment in comparison with values at the initial phase (p<0.05). However, 10 mg/d treatment group had no significant change in indexes before and after the treatment (p>0.05). Simultaneously, E, E/A ratio, LVEDD and LVEF was significantly higher whereas A was significantly lower in 40 mg/d treatment group than each corresponding values in 10 mg/d treatment group (p<0.05) at the terminal phase of this study, and these indexes in 40 mg/d treatment group also changed significantly after the treatment (p<0.05). There was no significant change in LVMI before and after the treatment both in 40 mg/d treatment group and 10mg/d treatment group, but the LVMI level in 40 mg/d treatment group decreased slightly compared with 10mg/d treatment group (Table-II).

***Comparison of additional medicines and adverse drug reactions during the follow-up period: ***During the follow-up period, there was no significant difference between two treatment groups (group A and group B) in the aspect of other medicine usage (p>0.05), these medicines including atorvastatin calcium tablets, aspirin enteric coated tablets, β-blocker, calcium antagonist, ACEI/ARB, nitrate ester, spironolactone tablets, diuretics and digoxin. One patient in each group received the hospitalized treatment due to the cardiac function deterioration. Neither of the two treatment groups had drug-induced hepatitis or rhabdomyolysis. Adverse drug reaction incidence between two treatment groups such as myalgia, polymyositis and gastrointestinal symptoms had no significant statistical difference (p>0.05).

## DISCUSSION

Myocardial ischemia, myocardial necrosis and myocardial remodeling after infarction are the main causes for heart failure. Oxidative stress can lead to lipid peroxidation, participate in the formation of oxidized low-density lipoprotein (ox-LDL), and trigger atherosclerosis. In particular, it can activate the cell apoptosis program, induce cardiac muscle cell death, reduce myocardial cells, and lead to different levels of heart dysfunction as a consequence. Statins can increase the expression of endothelial nitric oxide synthase (ENOS) and the stability of mRNA, which considerably reduce the occurrence of oxidative stress, also, improve the flow-mediated vasodilation of endothelial cells. Currently, statins have become a research focus because of its promising multifunctional effects such as anti-inflammatory, anti-oxidation and heart function improvement. Previous studies showed that continuous statins treatment could significantly improve the prognosis of ICM patients and congestive heart failure patients and extend their survival time.^2^ Statins’ treatment effects on cardiac failure are inconsistent so far, partially because of the disaccord of statins types, dosages and study population in different studies. This study adopted different doses atorvastatin treatment to ICM patients, to explore the statins' function and possible mechanism on ICM patients.

Oxidative stress is defined as the imbalance between the systemic manifestation of reactive oxygen species and a biological system's ability to readily detoxify the reactive intermediates or to repair the resulting damage; activated oxidative reaction would seriously imbalance the occurrence of free radical and antioxidant defense, and leads to tissue injury.^3^^,^^4^ The biological marker of oxidative stress is free radical. There are many sorts of free radicals but all with a short half-life period. Hence, it is quite difficult to test them directly. However, 8-OHdG, the oxidative product of the reaction between the free radical and DNA, protein, and lipid is more feasible to be detected. Under the reaction of DNA repair enzyme, 8-OHdG is released from the DNA, and goes into the blood circulation and is excreted in the urine, consequently, the level of 8-OHdG can be detected both in the blood serum and the urine, 8-OHdG has become the common biomarker in oxidative stress and DNA damage evaluation.^5^^,^^6^

Furthermore, oxidative stress plays an important role in the initiation and development of atherosclerosis, it is perceived as the main etiological factor that causes atherosclerosis, and has an important significance to monitor and evaluate the oxidative stress and for CHD's early detection and prevention. The oxidative stress can lead to lipoproteins oxygenizement, boost hypoxic cell damage, cell multiplication, inflammatory response and endothelial dysfunction, attack internal biomacromolecules such as DNA, lipid and protein, and form corresponding oxidative product, all of which participate in the initiation and development of atherosclerosis. 8-OHdG used to be studied in tumor or other diseases, however, recent research have found that, the 8-OHdG level in human atherosclerotic injury is high,^7^ as well as in DNA abstract from CAHD patients' peripheral blood lymphocyte. According to DNA damage evaluation of Botto et al.,^8^ it is demonstrated that compared with the control group, the DNA base damage level including 8-OHdG and DNA strand breaks level in CHD patients is elevated obviously, and there is a positive correlation between 8-OHdG level and the degree of coronary artery lesion. This study indicated that 8-OHdG level of ICM patients was (6.73±1.77) ng/ml, which was higher than normal control group (3.00±0.79) ng/ml, and the difference was statistically significant. Therefore, we feel that 8-OHdG level is crucially important in the development of ICM disease.

Previous studies indicated that dyslipidemia was the main risk factor of CHD and its complications. Statins has an effect of the cholesterol reduction, and can also decrease the vessel wall oxidative injury and inflammatory reaction level of CHD patients. Our study results showed that, as in the terminal phase, 8-OHdG, CRP, LDL-C and TC decreased slightly after the treatment, the 8-OHdG, CRP, LDL-C and TC in 40mg/d treatment group was obviously lower than 10 mg/d treatment group, the difference was statistically significant, and these indexes in 40 mg/d treatment group decreased significantly after treatment in comparison with values at the initial phase. However, 10 mg/d treatment group showed no significant change in previous indexes before and after the treatment. Therefore, we think that atorvastatin can not only reduce blood lipid, but also decrease the level of 8-OHdG and CRP, which indicates that atorvastatin has curative properties of blood lipid reduction and anti-ischemic effect through the mechanism of anti inflammatory,^9^^-^^11^ which holds similar viewpoint as previous studies. Concurrently, we have found that, dosage does affect the efficiency of blood lipids reduction and inflammatory reaction level reduction among CHD patients by atorvastatin treatment.

The dramatic decline of TC and LDL level assists the improvement of myocardial ischemia, endothelial function and capillary circulation in CHD patients, which finally improves their cardiac function. At the closing phase of this study, E, E/A ratio, LVEDD and LVEF in 40mg/d treatment group was significantly higher but BNP and A was significantly lower than 10 mg/d treatment group, and these indexes in 40 mg/d treatment group also changed significant after the treatment. Through the treatment, indexes which represent the cardiac systolic and diastolic functions were improved, whereas, there was no statistical significance before or after the treatment in 10 mg/d treatment group. Therefore, in our opinion, atorvastatin has the effect of improving heart function, and the effect also correlates with different doses. The left ventricular remodeling and geometric configuration alter is the key links of heart failure, currently LVMI is considered as an ideal indicator to reflect left ventricular hypertrophy. In this study, there was no significant change in LVMI before and after the treatment both in 40 mg/d treatment group and 10 mg/d treatment group, but the LVMI level in 40 mg/d treatment group decreased slightly compared with 10 mg/d treatment group, partly because of the short period of follow-up, so significant difference between two treatment groups couldn’t be observed.

In recent years, lots of researches have proved that in comparison with 10 mg/d atorvastatin treatment, 80 mg/d treatment could significantly reduce the risk of cardiovascular diseases and the incidence rate of vascular reconstruction, meanwhile without increasing the adverse drug reaction obviously.^12^ People in northern China have a relative heavier body weight, which makes them more likely to have fatty liver, and they have relatively lower tolerance to statins. So we adopted 40 mg/d atorvastatin in this study, during the follow-up period, there was no significant difference between two treatment groups in biochemical indicators such as ALT, creatine kinase and adverse drug reactions, which indicated that comparing with 10 mg/d atorvastatin group, 40 mg/d atorvastatin treatment had no obvious increase in adverse reactions.

**Table-I:** Comparison of general clinical characteristics in three groups ($\overset{\text{̅}}{x}$±s).

**Items**	**10 mg/d group** ** (n=62)**	**40 mg/d group** ** (n=61)**	**Control group** **(n=120)**
Age (years)	62.14±9.94	59.90±10.02	61.28±10.12
Hypertension	36(60.0%)	40(66.7%)	-
Diabetes	13(21.7%)	16(26.7%)	-
Coronary artery stent implantation	52(86.7%)	46(76.7%)	-
Cigarette smoke	10(16.7%)	13(21.7%)	24(20.0%)
Family history of CA	15(25.0%)	12(20.0%)	23(19.2%)
Blood uric acid (μmol/L)	333.62±160.88	325.08±162.95	319.92±165.21
Glycosylated hemoglobin (%)	5.56±1.74	5.34±1.36	5.08±1.09
TG (mmol/L)	1.73±0.78	1.60±0.62	1.52±0.56
HDL-C (mmol/L)	1.01±0.24	1.01±0.28	0.99±0.21
Cardiac function NYHA Ⅲ	56(93.3%)	54(90.0%)	-
Cardiac function NYHA Ⅳ	4(6.7%)	6(10.0%)	-
BMI (kg/m^2^)	25.1±2.6	24.4±2.8	23±2.5
8-OHdG (ng/ml)	6.79±1.60^a^	6.68±1.80^a^	3.00±0.79

a p<0.05, compared with control group.

**Table-II:** Comparison of indexes of laboratory and echocardiographic parameters in Group A and Group B at the initial and terminal phase of the study ($\overset{\text{̅}}{x}$±s).

**Items**	**10 mg/d group (n=62)**			**40 mg/d group (n=61)**	
	**Initial phase**	**Terminal phase**		**Initial phase**	**Terminal phase**
8-OHdG (ng/ml)	6.79±1.60	4.96±1.13		6.68±1.80	3.22±1.53 ab
CRP (ng/L)	10.7±2.04	9.30±0.91		9.9±1.01	5.9±0.31^a^^b^
BNP (pg/L)	623.22±254.22	488.22±149.79		674.44±243.29	312.70±175.92 ab
LDL-C (mmol/L)	3.58±1.51	2.99±1.25		3.53±1.21	2.10±1.31 ab
TC (mmol/L)	6.40±0.11	6.00±0.10		6.72±0.18	4.52±0.29 ab
CK (U/L)	98.58±27.31	101.65±39.76		92.66±31.61	98.91±41.87
ALT (U/L)	27.86±3.82	30.77±7.64		27.55±5.01	30.94±7.77
E (m/s)	0.54±0.07	0.60±0.09		0.53±0.06	0.79±0.11 ab
A (m/s)	0.68±0.11	0.61±0.09		0,.67±0.12	0.42±0.49 ab
E/A	0.70±0.11	1.22±0.13		0.7±0.10	1.91±0.08 ab
LVEDD (mm)	60.86±5.06	57.77±4.64		62.80±4.68	51.78±0.07 ab
LVESD (mm)	43.01±4.26	44.01±4.21		43.34±3.76	43.34±3.75
LVEF (%)	33.72±3.94	36.19±3.88		33.13±4.18	41.33±6.23 ab
LVMI (g/m^2^)	170.38±35.54	168.00±30.62		170.00±31.75	161.08±29.14

a p<0.05, comparison between 40 mg/d group and 10 mg/d group at the end of study;

b p<0.05, comparison before and after the treatment in 40mg/d group.

There are some limitations in this study. Firstly, the sample size is small which may affect the cogency of conclusion; secondly, this study lacks the contrast between different intensity of lipid-lowering statins, as well as too many study indexes and its variability might also influence the study results; thirdly, the imageological examination of left ventricular remodeling and geometric configuration is absent from this study which needs to be improved in further study.

## Author’s contributions

YJ conceived of the study and drafted the manuscript. CQ participated in the study design and coordination. YJ, QZ and LL helped to conceptualize the study, conducted the statistical analysis, and helped to draft the manuscript. ZL participated in the study design and contributed to the interpretation of results. YW oversaw the data collection and assisted in the implementation of the study. All authors contributed to critical revision of and approved the final manuscript.
